# Venous Thromboembolism after Community-Acquired Bacteraemia: A 20-year Danish Cohort Study

**DOI:** 10.1371/journal.pone.0086094

**Published:** 2014-01-23

**Authors:** Michael Dalager-Pedersen, Mette Søgaard, Henrik C. Schønheyder, Reimar W. Thomsen, John A. Baron, Henrik Nielsen

**Affiliations:** 1 Department of Infectious Diseases, Aalborg University Hospital, Aalborg, Denmark; 2 Department of Clinical Epidemiology, Institute of Clinical Medicine, Aarhus University Hospital, Aarhus, Denmark; 3 Department of Clinical Microbiology, Aalborg University Hospital, Aalborg, Denmark; 4 Department of Medicine, University of North Carolina School of Medicine, Chapel Hill, North Carolina, United States of America; D’or Institute of Research and Education, Brazil

## Abstract

**Background:**

Infections may increase the risk for venous thromboembolism (VTE), but little is known about VTE risk associated with community-acquired bacteraemia (CAB). We examined the risk for VTE within one year of CAB in comparison to that in matched controls.

**Methods:**

We conducted a population-based cohort study in North Denmark 1992–2011, using data from high-quality health-care databases. We included 4,213 adult CAB patients who had positive blood cultures drawn on the day of hospital admission, 20,084 matched hospitalised controls admitted for other acute medical illness, and 41,121 matched controls from the general population. We computed 0–90 and 91–365 day absolute risks for hospital-diagnosed VTE and used regression analyses with adjustment for confounding factors to compare the risk for VTE in bacteraemia patients and controls.

**Results:**

Among CAB patients, 1.1% experienced VTE within 90 days of admission and 0.5% during 91–365 days after admission. The adjusted 90-day odds ratio (OR) for VTE was 1.9 (95% CI 1.4–2.7) compared with hospitalised controls, and 23.4 (95% CI 12.9–42.6) compared with population controls. During 91–365 days after CAB admission, the VTE risk remained moderately increased (adjusted hazard ratio vs. hospitalised controls, 1.4; 95% CI 0.8–2.5, and vs. population controls, 1.9; 95% CI 1.0–3.3). Compared to hospitalised controls, the 90-day VTE risk increase was greater for Gram-positive infection (adjusted OR 2.5; 95% CI 1.6–4.1) than for Gram-negative infection (adjusted OR, 1.2; 95% CI 0.7–2.1), partly due to a high risk after *Staphylococcus aureus* infection (3.6%).

**Conclusion:**

The risk for VTE is substantially increased within 90 days after community-acquired bacteraemia when compared to hospitalised controls and population controls. However, the absolute risk of VTE following CAB is low.

## Introduction

Hospitalisations for community-acquired bacteraemia (CAB) have increased by 50% in recent years [1]. Venous thromboembolism (VTE) is a potentially life-threatening complication in hospitalised medical patients, and any association between CAB and VTE is therefore clinically important. The extent to which infections may cause VTE is debated. Previous observational studies have found 28-day or in-hospital risks of 0.5–1.5% for symptomatic VTE in patients hospitalised for sepsis or severe sepsis [2], [3] and a 1-year risk of 1.9% after hospitalisation for sepsis [2]. In acutely hospitalised medical patients, two studies have identified infection as an independent risk factor for VTE (odds ratio 1.27 and 1.74, vs. patients hospitalised for other disease) [4], [5]. Also, a recent case-control study found that patients hospitalised with infection had a 5- to 12-fold increased risk for VTE within 2 weeks after infection compared to population controls, and that this risk increase waned over time but remained significantly elevated for up to 1 year [6]. Cohort studies with long-term follow-up of VTE-risk after infection are sparse [2], [7], and none has included a comparison group. Previous studies have also lacked microbiological confirmation of infection, which may lead to a falsely inflated association due to possible misdiagnosis of VTE as skin infection or pneumonia.

We aimed to assess the short- and longer-term risks of symptomatic VTE in a cohort of hospitalised medical patients with microbiologically confirmed CAB compared with acutely hospitalised controls and with the background population.

## Materials and Methods

### Ethics Statement

This study was approved by the Danish Data Protection Agency (2011-41-5864). In accordance with Danish law, informed consent was not required for this study because it was entirely register-based.

### Design

We conducted this population-based cohort study in the North Denmark Healthcare Region, where all ∼500,000 inhabitants are provided with free tax-supported health care. All blood culture analyses in the Region are performed at the region’s referral hospital, Aalborg University Hospital, which provides inpatient care along with a dwindling number of district hospitals (from nine to five).

In Denmark, all residents are assigned a unique Civil Registration System (CRS) number which is registered in the CRS and in all medical registries. The CRS also stores information on date of birth, residency status, dates of immigration/emigration and death, updated daily. We used concatenated data from the CRS, the North Denmark Bacteraemia Database [8], the regional Hospital Discharge Registry (HDR) [9], and the Aarhus University Prescription Database [10].

For this study, we defined CAB as the presence of viable bacteria or fungi in the bloodstream isolated from blood cultures taken on the day of hospital admission, among patients who had not been admitted to hospital within the previous 30 days. The North Denmark Bacteraemia Database, which is described in detail elsewhere [8], was used for information on clinically relevant bacteraemia episodes, including date of blood culture draw, infectious agent, and focus of infection.

The HDR contains International Classification of Disease diagnosis code data (ICD, versions 8 and 10) on hospital discharges since 1977 and outpatient contacts since 1995 [9]. It allows for one primary diagnosis code (condition that prompted patient admission and the main condition responsible for the completed diagnosis and treatment course) and up to twenty secondary codes with no information as to which disease occurred first. We considered primary and secondary VTE codes from inpatient stays and hospital outpatient clinic visits as outcome events during 1992–2011. We specifically did not include VTE-diagnoses from emergency room visits because these codes may have a positive predictive value of only 31% [11]. The prescription registry details Anatomical Therapeutic Chemical Classification (ATC) codes on reimbursed prescriptions in the North Denmark region since 1989. We used the HDR and the prescription database for information on risk factors for VTE: cancer, cardiovascular disease, diabetes, obesity, chronic obstructive pulmonary disease, renal disease, recent hospital admission, recent surgery or trauma, and pregnancy [12]. The corresponding ICD and ATC codes can be found in Supplementary Table S1.

### Study Subjects

Eligibility criteria for all CAB patients and controls were age ≥15 years, residence in the study area for ≥1 year, no hospital stay in the previous 30 days, no history of bacteraemia, and no previous hospital contact with a VTE diagnosis.

We identified all eligible acutely ill medical (non-surgical) patients who had positive blood cultures taken on the day of admission during 1992–2010. To be confident that CAB preceded VTE in these cases, we first identified the patients (n = 26) who had been given a VTE code at discharge from a CAB hospitalisation since 1992. We then examined a convenience sample of cases with available medical files and discharge files, i.e. all cases since 1994 (n = 22, 85%). We found no indication of reverse causation in any patient. Seventeen patients developed VTE during their hospitalisation with CAB, and another five patients had had symptoms of infection for two to seven days before being diagnosed with both CAB and VTE on the day of admission.

Next, we identified two separate comparison cohorts. Because subsequent VTE risk may be related to acute medical hospital stay rather than to infection *per se*, we assembled a matched hospitalised comparison cohort of up to five acutely hospitalised patients for each CAB patient. As the CAB patients, hospitalised controls were admitted to medical departments, i.e. non-surgical and non-psychiatric departments. We excluded controls who were hospitalised because of VTE, i.e., who had a primary diagnosis of VTE from their index admission. The matching factors were sex, exact year of birth, and exact calendar year of hospital admission. Furthermore, for each CAB patient, we chose up to 10 eligible population controls, who were alive on the index date ( = date of hospital admission of the CAB patient), matched on sex and exact year of birth.

### Statistics

We followed all study subjects from the index date until first hospital encounter for VTE, death, emigration out of Denmark, or January 1^st^ 2012, whichever came first. We first computed absolute risks for VTE within 0–90 days and 91–365 days after the index date. Next, we used conditional logistic regression to compute odds ratios (ORs) with 95% confidence intervals (CIs) of VTE within 0–90 days and Cox proportional hazards models (stratified on matched groups) to compute hazard ratios (HRs) with 95% CIs of VTE during 91–365 days after the index date. Because VTE events were uncommon in CAB patients and controls during 0–90 days, odds ratios approximate relative risks and may be interpreted as such [13]. ORs and HRs for all CAB patients vs. matched controls were controlled for matching factors (age, gender, calendar-time) and further adjusted for cancer (yes/no), cardiovascular disease (yes/no), other comorbidity (diabetes, obesity, COPD, or renal disease - yes/no), and recent hospital contact (yes/no). Subgroup analyses were performed according to age group, gender, time period, causative pathogen(s), and focus of infection. These were controlled for matching factors and further adjusted for any comorbidity (cancer, cardiovascular disease, diabetes, obesity, COPD, or renal disease - yes/no).

Because the risk of VTE in the 90 days after CAB may be influenced more by the hospitalisation itself than by the CAB, in comparisons with the general population we also computed adjusted ORs of VTE during the CAB index admission vs. matched population controls experiencing VTE during 0–90 days (no population controls experienced VTE during the index admission of the matched CAB patient).

In supplementary analyses we first excluded the eight CAB patients (0.19%) who had a primary VTE diagnosis in the index admission, and their matched control groups. To examine the 90-day risk of VTE occurring clearly after admission/hospitalisation, we subsequently excluded CAB patients and hospitalised controls with any VTE code in the index admission. Finally, we computed the VTE risk after restriction to study subjects without recent surgery/trauma (previous 90 days) or hospital admission (previous 180 days), cancer history or new cancer in the following 365 days, or pregnancy in the 365 days surrounding the index date (here, termed “classic” risk factors for VTE).

The proportional hazards assumption was checked graphically by visual inspection of log- log plots. We used Stata 11.2 (Stata Corp., College Station, TX) for all analyses.

## Results

### Study Subject Characteristics

We identified 4,389 individuals with a first diagnosis of CAB, 21,626 matched acutely hospitalised controls, and 43,831 matched population controls. Among them, 176 CAB patients (4.0%), 709 hospitalised controls (3.3%), and 1,009 population controls (2.3%) had a previous diagnosis of VTE and were excluded. Table 1 shows baseline characteristics of the remaining 4,213 CAB patients and the matched hospitalised and population controls. The majority of study participants were women (53.5%) and the median age was 73 years (IQR 61–82). CAB patients and hospitalised controls had a similar burden of pre-existing disease (Supplementary Table S2). During the index hospitalisation, 10.9% of CAB patients had an intensive care unit stay as did 7.3% of hospitalised controls. Of note, mortality after CAB was 20.5% within 90 days and 29.3% within one year, whereas mortality was lower in both control groups (Table 1).

**Table 1:** Baseline characteristics, 90-day and 365-day mortality for study subjects, Northern Denmark, 1992–2010.

	Age, median (IQR)	Male(%)	PreviousCancer (%)	90-daymortality (%)	365-daymortality (%)
**All CAB patients (4,213)**	73.6 (61.2–82.2)	46.5	16.0	20.5	29.3
**All hospital controls (20,084)**	73.5 (60.9–82.1)	46.4	14.8	12.5	21.6
**All population controls (41,121)**	73.4 (60.9–82.0)	46.4	9.7	1.2	5.6
**CAB patients according to:**					
**Age, yrs**					
15–64 (1,306)	53.9 (42.2–59.9)	47.5	9.7	11.7	16.6
65–79 (1,568)	73.5 (69.8–77.1)	46.5	19.9	20.8	30.1
≥80 (1,339)	85.2 (82.5–88.5)	45.3	17.6	28.8	40.7
**Sex**					
Female (2,255)	73.8 (61.6–82.5)	-	15.8	19.6	27.1
Male (1,958)	73.3 (60.4–81.9)	-	16.2	21.6	31.8
**Study period**					
1992–2002 (2,134)	73.4 (60.7–81.7)	46.3	15.1	21.7	29.9
2003–2010 (2,079)	73.8 (61.4–82.7)	46.7	16.9	19.4	28.6
**Causative pathogen**					
Gram-positive (1,817)	70.8 (58.3–80.2)	51.9	15.2	22.0	30.1
* S. aureus* (302)	73.2 (61.7–81.3)	58.9	13.6	37.4	47.1
* S. pneumoniae* (994)	68.5 (55.8–78.9)	46.7	12.9	18.9	24.9
Other Gram-positive (521)	73.1 (61.5–81.6)	57.2	19.6	19.0	30.3
Gram-negative (2,120)	75.5 (63.7–83.0)	40.8	16.5	17.8	26.8
* E. coli* (1,468)	76.6 (66.3–83.6)	39.1	15.7	15.7	24.7
Other Gram-negative (652)	73.3 (56.8–81.7)	53.9	18.3	22.7	31.4
Polymicrobial/fungal (276)	78.4 (67.6–84.6)	54.4	17.8	31.5	43.1
**Focus of infection**					
Respiratory tract (939)	69.1 (55.9–79.2)	48.6	14.6	18.0	23.8
Urinary tract (1,382)	77.1 (66.4–83.7)	37.8	15.1	12.3	23.0
Skin, bone, or joint (253)	71.3 (60.6–79.2)	56.1	14.2	17.0	27.7
Miscellaneous (655)	69.7 (53.8–79.3)	51.1	14.4	20.9	27.5
Unknown or multiple (986)	76.4 (65.6–84.2)	51.1	20.4	35.8	45.8

Abbreviations: IQR, inter-quartile range.

### VTE Risk

For CAB patients, the 90-day absolute risk of VTE was 1.1% (Table 2), and the 91–365 day risk 0.5% (Table 3). Compared to hospitalised controls, CAB patients had a moderately increased risk of VTE during 0–90 days of follow-up (adjusted OR, 1.9; 95% CI 1.4–2.7; Table 2) and during 91–365 days (adjusted HR 1.4; 95% CI 0.8–2.5; Table 3). Compared to population controls, hospitalisation with CAB was associated with a greatly increased risk for VTE within 90 days (adjusted OR, 23.4; 95% CI 12.9–42.6; Table 2). Throughout the follow-up period most VTE events were diagnosed during a hospital stay (96.7% of events for CAB patients, 92.6% for hospitalised controls, and 86.7% for population controls).

**Table 2:** 0–90 day risk of a first VTE among patients with first hospital admission for CAB and matched hospitalised controls and population controls, Northern Denmark, 1992–2010.

		Risk, n/N (%)		Odds ratio (95% CI)^1^	Odds ratio (95% CI)^1^
	CAB patients	Hospitalised controls	Population controls	CAB vs. hospitalised controls	CAB vs. population controls
**All study subjects**	45/4,213 (1.1)	112/20,084 (0.6)	18/41,121 (0.0)	1.9 (1.4–2.7)	23.4 (12.9–42.6)
**Age group, years**					
15–64	15/1,306 (1.2)	27/6,307 (0.4)	2/12,936 (0.0)	2.7 (1.4–5.0)	102.0 (14.7–710.4)
65–79	25/1,568 (1.6)	47/7,460 (0.6)	8/15,305 (0.1)	2.6 (1.6–4.2)	29.7 (13.1–67.3)
≥80	5/1,339 (0.4)	38/6,317 (0.6)	8/12,880 (0.1)	0.7 (0.3–1.7)	6.3 (2.0–19.7)
**Sex**					
Male	26/1,958 (1.3)	45/9,322 (0.5)	11/19,073 (0.1)	2.8 (1.7–4.6)	21.5 (10.5–43.7)
Female	19/2,255 (0.8)	67/10,762 (0.6)	7/22,041 (0.0)	1.4 (0.8–2.3)	27.6 (11.3–67.4)
**Study period**					
1992–2002	23/2,134 (1.1)	43/10,260 (0.4)	7/20,905 (0.0)	2.6 (1.6–4.4)	29.3 (12.5–68.9)
2003–2010	22/2,079 (1.1)	69/9,824 (0.7)	11/20,216 (0.1)	1.5 (0.9–2.4)	19.7 (9.5–41.0)
**Causative pathogen**					
Gram positive	26/1,817 (1.3)	49/8,651 (0.6)	3/17,781 (0.0)	2.5 (1.6–4.1)	77.0 (23.2–255.8)
* S. aureus*	11/302 (3.6)	8/1,426 (0.6)	2/2,947 (0.1)	7.2 (2.7–19.2)	51.3 (11.3–232.3)
* S. pneumoniae*	7/994 (0.7)	30/4,751 (0.6)	0/9,738 (0)	1.1 (0.5–2.5)	-
Other Gram-positive	8/521 (1.5)	11/2,474 (0.4)	1/5,096 (0)	3.4 (1.4–8.4)	80.0^2^ (10.0–639.2)
Gram negative	14/2,120 (0.7)	58/10,115 (0.6)	14/20,653 (0.1)	1.2 (0.7–2.1)	9.8 (4.6–20.6)
* E. coli*	8/1,468 (0.5)	36/6,992 (0.5)	11/14,389 (0.1)	1.1 (0.5–2.3)	6.9 (2.8–17.4)
Other Gram-negative	6/652 (0.9)	22/3,123 (0.7)	3/6,364 (0)	1.4 (0.5–3.5)	20.0^2^ (5.0–80.0)
Polymicrobial and yeasts	5/276 (1.8)	5/1,318 (0.4)	1/2,687 (0)	5.0 (1.4–17.9)	49.2^2^ (5.7–421.1)
**Focus of infection**					
Urinary tract	7/1,382 (0.7)	33/6,582 (0.5)	12/13,431 (0.1)	1.3 (0.6–2.8)	7.4 (3.1–17.7)
Respiratory tract	5/939 (0.5)	28/4,491 (0.6)	0/9,180 (0)	0.8 (0.3–2.2)	-
Skin, bone or joint	13/253 (5.1)	8/1,197 (0.7)	1/2,491 (0.0)	8.8 (3.4–22.6)	124.3 (16.2–953.0)
Miscellaneous	7/695 (1.0)	16/3,341 (0.5)	1/6,929 (0.0)	2.1 (0.9–5.3)	69.1^2^ (8.5–561.4)
Unknown or multiple	11/944 (1.2)	27/4,473 (0.6)	3/9,313 (0.0)	1.8 (0.9–3.7)	17.7 (5.6–56.1)

Abbreviations: CI, confidence interval.

1 Computed by conditional logistic regression. Controls matched for age, sex and calendar time act as reference group. All odds ratio estimates are controlled for matching factors. Estimates for “All study subjects” are adjusted for cancer, cardiovascular disease, other comorbidity, and recent hospital contact. Subgroup analyses are adjusted for any comorbidity.

2 Not adjusted for comorbidity due to few events. A (−) denotes that the odds ratio could not be calculated because no events occurred among population controls.

**Table 3:** 91–365 day risk of first VTE diagnosis among patients with first hospital admission for CAB and matched hospitalised controls and population controls, Northern Denmark, 1992–2010.

		Risk, n/N (%)		HR (95% CI)^1^	HR (95% CI)^1^
	CAB patients	Hospitalised controls	Populationcontrols	CAB vs. hospitalised controls	CAB vs. population controls
**All study subjects**	15/3,316 (0.5)	45/13,920 (0.3)	72/32,022 (0.2)	1.4 (0.8–2.5)	1.9 (1.0–3.3)
**Age group, years**					
15–64	7/1,140 (0.6)	10/5,294 (0.2)	6/11,274 (0.1)	3.4 (1.2–9.9)	10.5 (3.4–32.6)
65–79	4/1,226 (0.3)	21/5,105 (0.4)	36/11,872 (0.3)	0.8 (0.3–2.4)	1.0 (0.4–2.9)
≥80	4/950 (0.4)	14/3,521 (0.4)	30/8,876 (0.3)	1.1 (0.4–3.6)	1.2 (0.4–3.3)
**Sex**					
Male	7/1,1519 (0.5)	21/6,313 (0.3)	30/14,628 (0.2)	1.5 (0.6–3.6)	2.0 (0.9–4.7)
Female	8/1,797 (0.4)	24/7,607 (0.3)	42/17,394 (0.2)	1.3 (0.6–3.0)	1.7 (0.8–3.8)
**Study period**					
1992–2002	4/1,658 (0.2)	15/6,929 (0.2)	27/16,066 (0.2)	1.1 (0.4–3.5)	1.2 (0.4–3.6)
2003–2010	11/1,658 (0.7)	30/6,991 (0.4)	45/15,956 (0.3)	1.5 (0.7–3.1)	2.3 (1.2–4.4)
**Causative pathogen**					
Gram positive	8/1,397 (0.6)	19/5,982 (0.3)	23/13,556 (0.2)	2.0 (0.8–4.7)	3.3 (1.5–7.5)
* S. aureus*	1/180 (0.6)	5/762 (0.7)	4/1,741 (0.2)	0.9^2^ (0.1–7.5)	2.4^2^ (0.3–21.9)
* S. pneumoniae*	4/802 (0.5)	7/3,489 (0.2)	9/7,798 (0.1)	2.8 (0.7–10.2)	4.2 (1.3–13.8)
Other Gram-positive	3/415 (0.7)	7/1,731 (0.4)	10/4,017 (0.2)	2.6 (0.6–11.7)	3.3 (0.8–12.8)
Gram negative	7/1,726 (0.4)	25/7,198 (0.3)	49/16,634 (0.3)	1.0 (0.4–2.5)	1.1 (0.5–2.5)
* E. coli*	5/1,233 (0.4)	16/5,066 (0.3)	34/11,828 (0.3)	1.3 (0.4–3.5)	1.2 (0.5–3.1)
Other Gram-negative	2/500 (0.4)	9/2,132 (0.4)	15/4,855 (0.3)	0.7 (0.1–3.4)	1.0 (0.2–4.4)
Polymicrobial and yeasts	0/186 (0)	1/740 (0.1)	0/1,783 (0)	-	-
**Focus of infection**					
Urinary tract	4/1,206 (0.3)	14/4,942 (0.3)	31/11,556 (0.3)	1.2 (0.4–3.8)	1.1 (0.4–3.1)
Respiratory tract	3/767 (0.4)	5/3,335 (0.1)	10/7,451 (0.1)	2.4 (0.5–10.6)	2.8 (0.8–10.4)
Skin, bone or joint	0/198 (0)	5/819 (0.6)	4/1,921 (0.2)	-	-
Miscellaneous	1/545 (0.2)	9/2,356 (0.4)	8/5,309 (0.2)	0.3 (0.0–2.6)	1.2 (0.1–10.3)
Unknown or multiple	7/600 (1.2)	12/2,468 (0.5)	19/5,785 (0.3)	2.6 (0.9–7.0)	3.1 (1.3–7.4)

Abbreviations: HR, Hazard Ratio. CI, confidence interval.

1 Computed by Cox regression stratified on matched groups. All hazard ratio estimates are controlled for matching factors (age, sex, calendar time). Estimates for “All study subjects” are adjusted for cancer, cardiovascular disease, other comorbidity, and recent hospital contact. Subgroup analyses are adjusted for any comorbidity.

2 Not adjusted for comorbidity due to few events. A (−) denotes that the hazard ratio could not be calculated.

Twenty-six CAB patients (0.6%) experienced VTE during the index admission. No population controls had a VTE diagnosis during the hospitalisation of the corresponding matched cases, but 18 (<0.1%) had a VTE during 0–90 days of follow-up, which corresponded to an adjusted OR of 13.9 (95% CI 7.2–26.8).

The VTE relative risk was especially elevated among young study subjects, both within 90 days and during 91 to 365 days after admission (vs. hospitalised controls, adjusted OR 2.7; 95% CI 1.4–5.0, and adjusted HR, 3.4; 95% CI 1.2–9.9) (Table 2 and Table 3).

Gram-positive CAB was associated with a higher relative risk for VTE vs. hospitalised controls within 0–90 days (adjusted OR, 2.5; 95% CI 1.6–4.1) than Gram-negative CAB (adjusted OR, 1.2; 95% CI 0.7–2.1) (Table 2). During 91–365 days of follow-up, Gram-positive CAB remained a high-risk infection (adjusted HR vs. hospitalised controls, 2.0; 95% CI 0.8–4.7; Table 3). Of note, patients with Gram-positive bacteraemia were ∼5 years younger than patients with Gram-negative bacteraemia (Table 1) but had a higher absolute 90-day risk of VTE (Table 2). In patients with Gram-positive CAB, a particularly high 90-day risk of VTE was found among patients with *S. aureus* infection (11/302, 3.6%), while the risk was lower in patients with *S. pneumonia* infection (7/994, 0.7%). Skin or bone/joint infection was also a high-risk infection for VTE (0–90 day risk for VTE of 5.1%, 13/253), and in this group the two most common infectious agents where *S. aureus* (0–90 day risk for VTE of 7.2%, 6/83) and β-hemolytic streptococci (0–90 day risk for VTE of 0.8%, 1/121).

After excluding CAB patients with a primary diagnosis of VTE at the index admission, CAB remained associated with an increased 90-day risk for VTE (OR vs. hospitalised controls, 1.6; 95% CI 1.1–2.3), see Supplementary Table S3. When examining the 90-day risk for VTE after index admission, CAB was associated with a 1.5-fold increased risk (OR) of VTE within 90 days when compared to hospitalised controls and a 9.2-fold increased risk (OR) when compared to population controls (Supplementary Table S3). In patients with no “classic” risk factors for VTE, CAB was associated with a 90-day OR of 2.8 when compared to hospitalised controls (95% CI 1.6–4.8; Supplementary Table S3).

## Discussion

In this population-based cohort study, we found that CAB was associated with a 1.1% risk of VTE within 90 days, nearly a doubling of the risk compared to other acutely hospitalised medical patients. Patients with gram positive bacteraemia, especially *S. aureus* bacteraemia, had a particularly high risk for VTE. When compared to matched hospitalised controls, *S. aureus* infection increased the 90-day risk for VTE by 600%.

This is the first study to examine the risk of VTE after microbiologically verified infection. It is also the largest cohort study to date to examine infection-related VTE risks. A few previous studies have detailed the absolute risk for VTE after infectious disease. In a cohort study of 1,080 hospitalised patients with presumed sepsis, 0.6% had VTE on admission, 1.3% developed in-hospital VTE and 0.6% suffered VTE between discharge and 1 year post-admission [2]. Similarly, pooled data on adverse events from three clinical trials showed a 0.5–0.9% 28-day risk for VTE in patients with severe sepsis [3]. Some clinical trials that specifically addressed the impact of heparin prophylaxis reported short-term absolute risk estimates for VTE ranging from 5 to 15% in medical patients hospitalised for acute infectious disease [5], [14]. The reason for this discrepancy is mainly that asymptomatic deep venous thromboses accounted for the majority of events in the trials.

Data on the relative risk increase for VTE conferred by infectious disease are sparse. In a Spanish registry-based study involving more than 1.5 million medical department discharges, acute infectious disease was associated with 1.27-fold increased risk for in-hospital VTE when compared to other diseases [4]. Using data from the MEDENOX trial, Alikhan et al. found that infectious disease was the only acute illness associated with a significantly increased risk for VTE in hospitalised medical patients (OR = 1.74 within 14 days, compared with other medical illness) [5]. To our knowledge only one previous study has examined how the risk for VTE after hospitalisation for infections varies according to the focus of the infection. In a case-control study, Schmidt et al. found an up to 12-fold increased risk for VTE within 2 weeks after hospitalisation for infections (vs. population controls), with the highest increase after skin infections [6]. Similarly, we found that microbiologically verified skin, bone and joint infections were high-risk infections for VTE, particularly if caused by *S. aureus.* That *S. aureus* skin and osteoarticular infection may be associated with a high risk for VTE has previously been suggested in case reports and smaller case series [15]–[17].

In a recent clinical guideline on the use of VTE prophylaxis in hospitalised medical patients, the American College of Physicians stated that a decision to initiate prophylactic heparin therapy should be based on an individualized assessment of the risk for VTE and bleeding, and that current evidence does not support the use of any specific VTE risk assessment tool [18]. Others have advocated the use of large observational datasets to identify inpatients who may benefit from VTE prophylaxis [19]. Our data indicate that it might be advantageous to include CAB and/or *S. aureus* infection in any future VTE risk assessment tool for use in medical inpatients.

Infections may induce thromboembolism by a number of mechanisms [20]. During systemic inflammatory activity, endothelial cell apoptosis, tissue-factor expression, thrombin generation and fibrin deposition is increased, while anticoagulatory pathways and fibrinolysis are impaired [20], [21]. Gram-positive bacteria, including *S. aureus*, may have an exceptionally high propensity for inducing thrombosis [17], [22]. Immobilization is a risk factor for VTE [12] and is pronounced during severe infection, especially infection causing bone and joint pain [23].

Strengths of the present study include the large sample size and microbiological verification of infection. We used computerized medical databases of high quality and validated VTE codes [8], [11]. Furthermore, we had complete and long follow-up.

However, this study also has limitations. The predictive value of a VTE discharge code (from wards including outpatient clinics) in the Danish registries approaches only 75% [11], which would bias our estimates towards the null if misclassification was similar in CAB patients and controls. The 90-day mortality among CAB patients was 20.5% in our study, and it could be argued that some patients may have died from an unidentified pulmonary embolism, which may have led to an underestimation of the true pulmonary embolism risk after CAB. Surveillance bias is possible, particularly for infections that clinically mimic VTE. However, pneumonia and skin infections caused by β-hemolytic streptococci were associated with a relatively low absolute risk of VTE, indicating that this bias is likely to be small if present at all. There is a risk that VTE may have preceded CAB in some patients. To decrease the risk for reverse causation, we restricted the study to patients who had positive blood cultures on the day of admission. A further argument against reverse causation is that our review of a sample of medical records did not reveal instances in which the VTE preceded the CAB. Another issue that should be taken into consideration is the lack of data on in-hospital medication use. For instance, anticoagulant use could have lowered the risk of VTE and we may therefore have underestimated the VTE risk increase associated with bacteremia in the absence of this treatment, particularly versus population controls. We used information from health-care databases to adjust for important risk factors for VTE, but residual and unmeasured confounding remain possible, for example thrombophilia.

We conclude that CAB is associated with a substantially increased short-term risk for VTE. However, with the possible exception of *S. aureus* infection, the absolute risk for VTE after CAB is low.

## Supporting Information

Table S1 **ICD and ATC codes.**
(DOCX)

Table S2 **Descriptive characteristics of 4,213 patients admitted with a first diagnosis of community-acquired bacteraemia and their matched controls, 1992–2010.**
(DOCX)

Table S3 **Risk of VTE in CAB patients and controls when restricting analysis to patients with no VTE diagnosis during index admission and to patients with no “classic” risk factor for VTE.**
(DOCX)
