# Supplementary material for: Venous Thromboembolism after Community-Acquired Bacteraemia: A 20-year Danish Cohort Study
Source: PLoS One. 2014 Jan 23;9(1):e86094. doi: 10.1371/journal.pone.0086094 (PMC3900448; doi:10.1371/journal.pone.0086094)
Supplement: Table S1 — ICD and ATC codes. (DOCX) [file pone.0086094.s001.docx]

| **Outcome** | **ICD codes** |
| --- | --- |
| **Deep venous thrombosis** | ICD-8: 451.00, 451.08, 451.09, 451.90, 451.92, 451.99, 452-453, ICD-10: I80.1-9, I81-82 |
| **Pulmonary embolism** | ICD-8: 450.99, ICD-10: I26 |
| **Comorbidities (previous) and pregnancy** | **ICD and ATC codes^1^** |
| **Cardiovascular disease** |  |
| Myocardial infarction | ICD-8: 410; ICD-10: I21-I23 |
| Cerebrovascular disease | ICD-8: 430-438; ICD-10: I60-I69, G45-G46 |
| Congestive heart failure | ICD-8: 427.09, 427.10, 427.11, 427.19, 428.99, 782.49, ICD-10: I11.0, I13.0, I13.2, I50 |
| Peripheral vascular disease | ICD-8: 440-445; ICD-10: I70- I74, I77 |
| ACE inhibitors | ATC: C09 (C02 before 1 January 1996) |
| Beta blockers | ATC: C07 |
| Calcium channel blockers | ATC: C08 |
| Diuretics | ATC: C03 |
| Nitrates | ATC: C01DA (if ≥2 prescriptions are registered) |
| Aspirin | ATC: B01AC06, N02BA01 (in previous 125 days) |
| **Diabetes** | ICD-8: 249, 250; ICD-10: E10-E11; ATC: A10 |
| **Chronic pulmonary disease** | ICD-8: 490-493, 515-518; ICD-10: J40-J47, J60-J67, J68.4, J70.1, J70.3, J84.1, J92.0, J96.1, J98.2, J98.3; ATC: R03 |
| **Renal disease** | ICD-8: 403, 404, 580-583, 584, 590.09, 593.19, 753.10-753.19, 792; ICD-10: I12, I13, N00-N05, N07, N11, N14, N17-N19, Q61 |
| **Cancer** | ICD-8: 140-209; ICD-10: C00-C97 |
| **Obesity** | ICD-8: 277; ICD-10: E65-E68 |
| **Trauma** | ICD-8: 800.09-959.99; ICD-10: S00-T14 |
| **Pregnancy** | ICD-8: 630-680; ICD-10: O00-O99 |

^1^Drugs are any previous use, unless otherwise specified.
