# Supplementary material for: Venous Thromboembolism after Community-Acquired Bacteraemia: A 20-year Danish Cohort Study
Source: PLoS One. 2014 Jan 23;9(1):e86094. doi: 10.1371/journal.pone.0086094 (PMC3900448; doi:10.1371/journal.pone.0086094)
Supplement: Table S2 — Descriptive characteristics of 4,213 patients admitted with a first diagnosis of community-acquired bacteraemia and their matched controls, 1992–2010. (DOCX) [file pone.0086094.s002.docx]

|  | **Community-acq. bacteraemia patients**  **(n=4,213)** | **Acutely**  **hospitalised controls**  **(n=20,084)** | **General**  **population controls**  **(n=41,121)** |
| --- | --- | --- | --- |
| **Age, yrs** |  |  |  |
| 15-64 | 1,306 (31.0) | 6,307 (31.4) | 12,936 (31.5) |
| 65-79 | 1,568 (37.2) | 7,460 (37.1) | 15,305 (37.2) |
| ≥80 | 1,339 (31.8) | 6,317 (31.5) | 12,880 (31.3) |
| **Sex** |  |  |  |
| Female | 2,255 (53.5) | 10,762 (53.6) | 22,048 (53.6) |
| Male | 1,958 (46.5) | 9,322 (46.4) | 19,073 (46.4) |
| **Comorbidity** |  |  |  |
| Cancer | 674 (16.0) | 2,980 (14.8) | 3,988 (9.7) |
| Cardiovascular disease^1^ | 3,041 (72.2) | 13,044 (65.0) | 23,005 (55.9) |
| Chronic pulmonary disease^1^ | 1,457 (34.6) | 6,226 (31.0) | 8,903 (21.7) |
| Diabetes mellitus^1^ | 645 (11.1) | 2,023 (10.1) | 2,594 (6.3) |
| Obesity | 222 (5.3) | 705 (3.5) | 730 (1.8) |
| Renal disease | 142 (3.4) | 476 (2.4) | 430 (1.1) |
| **Recent hospital contact** |  |  |  |
| Inpatient admission^2^ | 946 (22.5) | 2,643 (13.2) | 3,136 (7.6) |
| Surgery^3^ | 542 (12.9) | 2,842 (14.2) | 1,654 (4.0) |
| Trauma^3^ | 174 (4.1) | 947 (4.7) | 580 (1.4) |
| Pregnancy^4^ | 5 (0.1) | 79 (0.4) | 148 (0.4) |

Data are no. (%) of patients.

^1^Includes previous use of drugs for cardiovascular disease, chronic pulmonary disease and diabetes, respectively. ^2^Inpatient hospital admission within 180 days before the index date. ^3^Any surgery or trauma within 90 days before the index date. ^4^Pregnancy within 365 days of the index date.
