# Supplementary material for: Venous Thromboembolism after Community-Acquired Bacteraemia: A 20-year Danish Cohort Study
Source: PLoS One. 2014 Jan 23;9(1):e86094. doi: 10.1371/journal.pone.0086094 (PMC3900448; doi:10.1371/journal.pone.0086094)
Supplement: Table S3 — Risk of VTE in CAB patients and controls when restricting analysis to patients with no VTE diagnosis during index admission and to patients with no “classic” risk factor for VTE. (DOCX) [file pone.0086094.s003.docx]

|  | **0-90 day risk in patients with no primary diagnosis of VTE from index admission^2^** | **0-90 day risk in patients with no diagnosis of VTE from index admission** | **0-90 day risk in patients with no “classic” risk factor^1^** | **91-365 day risk in patients with no “classic” risk factor^1^** |
| --- | --- | --- | --- | --- |
| **Absolute risk, n/N (%)** |  |  |  |  |
| CAB patients | 37/4,205 (0.9) | 19/4,187 (0.5) | 26/2,426 (1.1) | 9/2,008 (0.4) |
| Hospitalised controls | 112/20,046 (0.6) | 62/19,908 (0.3) | 28/7,274 (0.4) | 14/5,575 (0.3) |
| Population controls | 18/41,041 (0.0) | 18/40,867 (0.0) | 9/19,251 (0.0) | 26/15,920 (0.2) |
| **Adjusted relative risk (95% CI)^3^** |  |  |  |  |
| CAB patients vs. hospitalised controls | 1.6 (1.1-2.3) | 1.5 (0.9-2.5) | 2.8 (1.6-4.8) | 1.7 (0.7-4.0) |
| CAB patients vs. population controls | 18.0 (9.7-33.4) | 9.2 (4.5-18.5) | 21.9 (10.1-47.6) | 2.2 (1.0-4.8) |

Abbreviations: CAB, community-acquired bacteremia. CI, confidence interval. ^1^No recent surgery/trauma (previous 90 days) or hospital admission (previous 180 days), cancer history or new cancer in the following 365 days, or pregnancy in the 365 days surrounding the index date. ^2^Secondary VTE diagnoses are included. ^3^Odds ratio computed using conditional logistic regression (0-90 day risk) and hazard ratio using Cox regression (91-365 day risk), controlled for age, sex, calendar-time, and further adjusted for cardiovascular disease, and other comorbidity (diabetes, obesity, COPD, renal disease). Analyses in patients with no diagnosis of VTE from index admission are also adjusted for cancer and recent surgery/trauma/admission.
